# Seroprevalence of Hepatitis C, Hepatitis B, Cytomegalovirus, and Human Immunodeficiency Viruses in Multitransfused Thalassemic Children in Upper Egypt

**DOI:** 10.1155/2016/9032627

**Published:** 2016-02-17

**Authors:** Ramadan A. Mahmoud, Abdel-Azeem M. El-Mazary, Ashraf Khodeary

**Affiliations:** ^1^Department of Pediatrics, Faculty of Medicine, Sohag University, Sohag 82524, Egypt; ^2^Department of Pediatrics, Faculty of Medicine, Minia University, Minia 61111, Egypt; ^3^Department of Clinical Pathology, Faculty of Medicine, Sohag University, Sohag 82524, Egypt

## Abstract

*Background*. Frequent blood transfusions in thalassemia major children expose them to the risk of transfusion-transmitted infections (TTIs). The aim of this study was to estimate the prevalence of hepatitis C virus (HCV), hepatitis B virus (HBV), human immunodeficiency virus (HIV), and cytomegalovirus (CMV) in thalassemic children attending the Pediatrics Departments of both Sohag and Minia Universities of Upper Egypt, during the period from May 2014 to May 2015.* Methods*. Serum samples were screened for hepatitis B surface antigen (HBsAg), anti-HCV, anti-CMV, and anti-HIV type 1 and type 2 using the Vitek Immunodiagnostic Assay System.* Results*. The frequencies of anti-HCV, HBsAg, anti-CMV, and anti-HIV type 1 and type 2 were found to be 37.11%, 4.12%, 4.12%, 0.00%, and 0.00%, respectively. Seropositivity for anti-HCV, HBsAg, and anti-CMV increased with increasing age of the patients, duration of the disease, serum ferritin level (ng/mL), and liver enzymes (U/L), while it was not significantly associated with gender, frequency of blood transfusion, or the status of splenectomy operation (*P* > 0.05).* Conclusion*. The frequency of TTIs, especially HCV, is considerably high among Egyptian children with thalassemia major. It is therefore important to implement measures to improve blood transfusion screening, such as polymerase chain reaction, in order to reduce TTIs from blood donor units.

## 1. Introduction

Thalassemias are the commonest monogenic disorders in the world [1], and the incidence rate is higher in the Middle East [2]. *β*-thalassemia constitutes a major health problem in Egypt with an estimated carrier rate of 9-10% [3]. It is an autosomal recessive disorder of hemoglobin synthesis caused by a direct downregulation in the synthesis of structurally normal *β*-globin chains. Due to the excess *α*-globin chains relative to the *β*-globin chains, *α*-globin tetramers (*α*4) are formed and interact with the red cell membrane, leading to hemolytic anemia and increased erythroid production [4].

Survival of the patients mainly depends upon regular blood transfusions, which may lead to further complications, such as absorptive iron overload and transfusion-transmitted infections (TTIs), and this may contribute to the morbidity and mortality of patients with thalassemia major [5].

Screening of donor blood through national protocols for possible infections, including hepatitis B virus (HBV), hepatitis C virus (HCV), cytomegalovirus (CMV), and human immunodeficiency virus (HIV), is considered the optimal preventive method. There is a constant need to explore the effect of currently used protocols of blood donor screening by determining the burden of TTIs in multitransfused patients [6].

In Egypt the lack of a central surveillance system for disease epidemiology is a major obstacle regarding the assessment of the current situation of infectious diseases, including TTIs, and, therefore, regional and periodic studies are the only option to monitor recent developments. There are only a few studies about TTIs in thalassemic children in Egypt [3, 7–9]. To our knowledge, no study has described the situation of TTIs in thalassemic children in Upper Egypt. These areas had low social economic income compared to other parts in Egypt [10]. The objective of this study was to evaluate the seroprevalence of HBV, HCV, CMV, and HIV. Furthermore, TTI-associated clinical and laboratory risk factors were investigated in this study including gender, family history, duration of the illness, frequency of blood transfusions, splenectomy, hemoglobin, ferritin level, creatinine levels, and liver enzymes.

## 2. Materials and Methods

In this cross-sectional study, we analyzed blood samples taken from 97 transfusion-dependent thalassemic children during the period from May 2014 to May 2015 in the Pediatrics Departments, Faculty of Medicine, in both Sohag and Minia Universities of Upper Egypt. Ethical approval for the study was obtained from the Research Committee of the Medical Faculty, Sohag and Minia Universities, and written informed consents were obtained from all guardians/parents of the children prior to data and sample collection.

Inclusion criteria included all known cases of *β*-thalassemia major according to hemoglobin electrophoresis data. Complete information regarding clinical profile and family history of patients was also mandatory for inclusion criteria.

Hemophilic children, as well as children with other types of hemolytic anemias, such as *α*-thalassemia, sickle cell anemia, and spherocytosis were excluded from the study.

Thalassemic children were subjected to a detailed history and thorough clinical examination. Special emphasis was given on personal history (age, gender, and location), family history (parent consanguinity, and family history of similar conditions), clinical data (age of diagnosis and number of blood transfusions), and clinical examination (pallor, jaundice, hepatomegaly, cirrhotic manifestations, splenomegaly, splenectomy, and murmur on the heart) and laboratory data (hemoglobin, serum ferritin levels, serum creatinine, and liver enzymes) were recorded.

For laboratory assessments, 7 mLs of blood was obtained by venipuncture with strict sterile measures into a sterile plane tube and pediatric EDTA vacationer tube. In children receiving blood transfusions, samples were drawn before packed-RBC transfusion. Serum was collected in two tubes: one for serological testing and the other for the HCV-RT-PCR. The serum was kept at −20°C until the time of the assay.

For the serological tests, HBsAg, HCV antibodies, CMV IgM antibodies, and HIV type 1 and type 2 antibodies were measured by the Vitek Immunodiagnostic Assay System (VIDAS-BioMerieux, France) which is an enzyme-linked fluorescent assay, and it was performed according to the manufacturer's instructions. Briefly, the instrument uses a disposable pipette tip called the solid-phase receptacle, which is coated with antigens and also acts as a pipetting device. All the ready-to-use reagents are contained in a sealed strip. The specimen (serum or plasma) is added to the reagent strip, and all the following steps of the test are done automatically, without any further manipulation.

The positive HCV cases were confirmed and tested for viral load by reverse transcriptase polymerase chain reaction (RT-PCR) [11]. RNA extraction was carried out using the Qiagen RNA extraction kit according to the kit manual. PCR was performed using the Applied Biosystems® TaqMan® Universal PCR master mix and the Applied Biosystems StepOne*™* Real-Time PCR System according to the manufacturer's instructions.

A complete blood count (CBC) was performed with all samples using a Celtic autocounter after calibration. Pretransfusion samples were considered for patients requiring blood transfusions at the time of study. Serum ferritin levels, alanine transaminase level (ALT), aspartate aminotransferase (AST), and creatinine levels were measured using VIDAS.

### 2.1. Statistical Analysis

Data was analyzed using SPSS (Statistical Package of Social Science) version 16. Quantitative data are represented as the mean (standard deviation) or median (range). Data were analyzed using the Mann-Whitney test as they were not normally distributed. Qualitative data are presented as number and percentage and were compared using either the Chi-square test or the Fisher exact test. Graphs were produced using Excel software. The *P* value was considered significant at *P* < 0.05.

## 3. Results

In the study with a total of 97 thalassemic children, 36.08% of patients were female and 63.92% were male. The mean age at the time of study was 8.89 (5.07) years (range: 6–18 years). The mean age at diagnosis was 11.40 (13.18) months. The mean duration of the disease was 7.94 (4.90) years. The mean ferritin level was 2875 (1764) (ng/mL). Complete history and clinical examination was done for all the children included in the study as shown in Table 1.

A total of 36 patients (37.11%) were found to be anti-HCV antibody- (anti-HCV-) positive (Table 2). All positive cases were confirmed by HCV-RT-PCR. There were only 4 (4.12%) patients that were found to be positive for HBsAg and anti-CMV IgM. There were no cases that were positive for anti-HIV type 1 or type 2. Three patients had two infectious diseases simultaneously (two had HBV and HCV, and one had HCV and CMV).

The risk factors for hepatitis C virus are shown in Table 3 and include age of the patients [7.69 (5.01) years in negative cases compared to 12.63 (4.96) years in positive cases (*P* = 0.04)], mean ferritin level [2416 (1283) ng/mL in negative cases compared to 3652 (2173) ng/mL in positive cases (*P* = 0.006)], serum ALT [48 (7.4) U/L in negative cases compared to 65 (5.1) U/L in positive cases (*P* = 0.001)], serum AST [47 (9.44) U/L in negative cases compared to 61 (8.9) U/L in positive cases (*P* = 0.02)], and duration of the disease [7.75 (4.62) years in negative cases compared to 10.56 (5.48) years for positive cases (*P* = 0.02)]. With a total of 97 patients included in the study there were 6 patients (6.18%) who had manifestations of liver cirrhosis, two were HBV and HCV positive, two were HCV positive, and two were serologically free of viruses.

For hepatitis B virus infection, risk factors are shown in Table 4 and included the age of patients [7.77 (4.08%) years in negative cases compared to 13.5 (1.73) years in positive cases (*P* = 0.04)], mean ferritin level [2809 (1750) ng/mL in negative cases compared to 4407 (1541) ng/mL in positive cases (*P* = 0.04)], serum ALT [43 (4.4) U/L in negative cases compared to 58 (3.5) U/L in positive cases (*P* = 0.03)], serum AST [42 (9.44) U/L in negative cases compared to 63 (6.3) U/L in positive cases (*P* = 0.04)], and duration of the disease [7.71 (4.86) years in negative cases compared to 13.25 (1.73) years in positive cases (*P* = 0.02)].

CMV risk factors are shown in Table 5 and include age of the patients [8.69 (6.01) years in negative cases compared to 13.63 (4.21) years in positive cases (*P* = 0.03)], mean ferritin level [2894 (1770) ng/mL in negative cases compared to 4735 (1789) ng/mL in positive cases (*P* = 0.04)], and mean duration of the disease [7.82 (4.92) years in negative cases compared to 13.69 (3.79) years in positive cases (*P* = 0.03)]. The distribution of disease status (thalassemia and hepatitis B, hepatitis C, CMV, and HIV) was not significantly associated with gender, frequency of blood transfusion, hemoglobin level, creatinine level, or splenectomy operations (*P* > 0.05).

## 4. Discussion

Thalassemia major is one of the most common autosomal single-gene thalassemic disorders. It is prevalent in more than 60 countries of the world with a carrier population of up to 150 million worldwide [12]. In order to reduce the complications of severe anemia in *β*-thalassemic patients, early and regular blood transfusion therapy is mandatory. Thalassemic patients had a risk for iron overload and for TTIs, such as HBV, HCV, CMV, and HIV [13]. The current study focuses on the prevalence of these viruses in children attending the Pediatrics Departments of both Sohag and Minia Universities of Upper Egypt.

In Egypt, the screening tests for the blood donors include HCV antibodies, HBsAg, and HIV antibodies. Moreover, in the 2000 some blood bank added CMV antibody and syphilis antibodies to the blood donor screening [8]. These tests were done by ELISA or immunofluorescence based methods in national and governmental blood banks which have high sensitivity and specificity [14]. However, many nongovernmental and private hospitals sometimes used rapid and cheap immunochromatographic methods for blood donors screening which have lower sensitivity and specificity, which contribute to the increased rates of TTIs [15]. Furthermore, the diagnosis of viral hepatitis in the window phase is still an obstacle in Egypt, because of Egypt's constrained economy which limits the implementation of sensitive screening techniques as RT-PCR for all cases [16].

Egypt has one of the highest prevalence rates of the virus in the world; an estimated 10–15% of the population, about 8–10 million people, is carrying hepatitis C antibodies. Five million of those are actively infected. The iatrogenic role of parenteral antischistosomal therapy campaigns carried out until the 1980s may have contributed to such high incidence of HCV. Sharing razors, piercing, and tattooing were also suggested as possible explanations [17].

The implementation of routine blood donor screening tests for HCV in the early 1990s led to a reduction in the transfusion of HCV to blood recipients [18], from about 70% in one cohort study [19] to 37% in this present study. Furthermore, the introduction of advanced screening methods, such as RT-PCR, has led to a further decline in transfusion-related viral hepatitis [11]. RT-PCR technologies can detect viremia earlier than the existing screening tests. Blood centers in some developed countries as the United States have implemented RT-PCR routinely for all blood donations since 1999. This strategy has reduced the window period for HCV detection by 50–60 days and decreased the risk for HCV transmission from 1 in 103,000 to 1 in 2,000,000 transfused units [20].

However, despite these efforts, posttransfusion transmission of HCV has remained a major health problem in multitransfused patients, leading to increased morbidity and mortality. In the present study, more than 37% of patients were positive for anti-HCV. These results can be compared with previously conducted studies from other regions of Egypt, such as anti-HCV in one Egyptian report of about 34% [7], and in another study of about 24% [8]. Moreover, in other countries, anti-HCV-positive cases were about 40.5% in Jordan [21], 30% in India [22], and 35% in Pakistan [23].

Furthermore, the severity of liver cirrhosis due to HCV in patients with thalassemia may be greater because of concomitant iron overload. It has been demonstrated that iron and HCV infection are independent but mutually reinforcing risk factors for the development of liver fibrosis and cirrhosis. It appears therefore that patients with thalassemia, particularly those with poor control of iron overload, face an increased risk of developing cirrhosis [24].

In the present study, four patients were HCV positive and had manifestations of liver cirrhosis. Ardalan et al. found that the rate of liver fibrosis accelerated in patients with iron overload and HCV when compared to patients with thalassemia major alone [25]. Moreover, Zurlo et al. found that advanced liver fibrosis is one of the most common causes of death in transfusion-dependent thalassemia patients over 15 years old [26].

Children with thalassemia major are particularly susceptible to HBV because they receive multiple blood transfusions. These children have higher infection rates than normal children. Egypt has adopted a universal hepatitis B vaccine service for infants since 1992. The primary objective of vaccination is to eliminate chronic HBV infections and ultimately reduce the reservoir for new infections [17]. In the present study, about 4% were positive for HBV, and in another Egyptian study [8], about 3% were positive for HBV. These results were slightly higher than a recent North Indian study, in which out of the 462 thalassemic children 13 cases (2.8%) were positive for HBsAg by ELISA [27], and also higher than an Iranian study in which only 1.5% were HBsAg positive [28].

Screening in most Egyptian blood banks is performed for HBsAg but not for core antibody (anti-HBc). Anti-HBc appears at the onset of acute HBV infection and may also indicate chronic infection. The routine implementation of anti-HBc testing was recommended to reduce the risk of transfusion-transmitted HBV. In a previous Egyptian study, 7.8% of blood samples were anti-HBc positive, of which 6.25% were HBV DNA positive [29]. In another Egyptian study by Said et al., they found that the prevalence of anti-HBc among HBsAg negative blood donors was 14.2% [30]. Furthermore, HBV can also be transmitted through components that are HBsAg negative but HBV DNA positive. Altindis et al. recommended routine screening of blood units by sensitive PCR-based methods, to detect possible occult HBV infections [31].

For CMV, only 4% of thalassemic patients were CMV positive, and these results were slightly higher than the Jamal et al. study in Malaysia [32], which found that the incidence of CMV was about 1.8%. Transfusion-transmitted HIV is, fortunately, not a major cause for concern, owing to advanced testing techniques, such as the recent generation of ELISA screening tests, as well as decreasing prevalence of such diseases, which is less than 0.02% in Egyptian population [33], conservative culture, and increasing awareness about this infection in the general population. Therefore, in this study, we did not find any positive cases for HIV type 1 or type 2. These results corroborate other studies [28, 32] that also found no positive cases for HIV in thalassemic patients. Nevertheless, an Indian study found that the seroprevalence of HIV was about 1.5% [34].

As shown in the present study, patients with thalassemia have a high prevalence of hepatitis B and hepatitis C infections. Furthermore, HCV and HBV infected patients had significantly higher liver enzymes than noninfected patients. Chronic hepatitis C virus infection has been associated with liver iron loading. The cause of elevated serum iron indices in some HCV-infected individuals is not clear. The concomitant increase of serum AST and ALT levels suggests that iron and ferritin are released from damaged hepatocytes as a result of hepatic necroinflammation [35]. In addition, increased iron has been shown to enhance HCV replication in vitro [36].

In the current study, splenomegaly was observed in 96.9% of patients, whereas 90.73% of patients had hepatomegaly. The distribution of HCV, HBV, CMV, and HIV was studied by considering different clinical parameters related to thalassemia. In this study, we found that older age of the patients, longer duration of the disease in years, elevated liver enzymes (U/L), and increasing ferritin level (ng/mL) are associated with higher seroprevalence of TTIs. Similar findings were reported by other studies [37–39]. Though the standardized blood screening procedures were outlined in the 1990s for blood-related products and were subsequently implemented in various countries, higher prevalence of TTIs, especially HCV, among thalassemic patients requires greater attention from a public health perspective. Screening of blood donors by advanced ELISA screening tests and RT-PCR could detect HCV in early stages of diseases and will provide better opportunities for risk assessment.

## 5. Conclusion

A total of 97 homozygous *β*-thalassemia patients were included in this study. The seroprevalence of HCV was the highest TTI in thalassemic children, with a lower percentage for HBV and CMV cases but there were no HIV cases. These seroprevalence-positive cases were significantly associated with older age, longer duration of diseases, increased liver enzymes, and higher ferritin levels. Recent solid-blood screening programs including advanced ELISA screening tests, liver enzymes, and RT-PCR will hopefully reduce the risk of TTIs associated with blood transfusions.

## Figures and Tables

**Table 1:** Demographic characteristics of beta thalassemia major children.

Characteristics		Summary statistics
Age/years	Mean (SD)	8.89 (5.07)
	Median (range)	10 (6–18)

Gender	Females	35 (36.08%)
	Males	62 (63.92%)

Age at diagnosis/months	Mean (SD)	11.40 (13.18)
	Median (range)	6 (3–72)

Frequency of blood transfusion/months	Mean (SD)	1.09 (0.42)

Duration of disease/years	Mean (SD)	7.94 (4.90)

Family history	No	42 (43.30%)
	Yes	55 (56.70%)

Ferritin level (ng/mL)	Mean (SD)	2875 (1764)

Alanine transaminase (ALT) (U/L)	Mean (SD)	55 (15.4)

Aspartate aminotransferase (AST) (U/L)	Mean (SD)	62 (9.44)

Creatinine (mg/dL)	Mean (SD)	0.6 (0.54)

Hb level (g/dL)	Mean (SD)	6.77 (1.87)

Hepatomegaly	No	9 (9.27%)
	Yes	88 (90.73%)

Splenomegaly	No	3 (3.09%)
	Yes	94 (96.9%)

Splenectomy	No	56 (57.73%)
	Yes	41 (42.27%)

Age at splenectomy/years	Mean (SD)	8.39 (2.47)

Pallor	No	11 (11.34%)
	Yes	86 (88.66%)

Jaundice	No	80 (82.47%)
	Yes	17 (17.53%)

Murmur	No	56 (57.73%)
	Yes	41 (42.27%)

**Table 2:** Prevalence of transfusion transmitted infections (TTIs) in thalassemic children.

Transfusion transmitted infections (TTIs)		Numbers (percentage)
Hepatitis C virus	Negative	61 (62.89%)
	Positive	36 (37.11%)

Hepatitis B virus	Negative	93 (95.88%)
	Positive	4 (4.12%)

CMV	Negative	93 (95.88%)
	Positive	4 (4.12%)

HIV	Negative	97 (100%)

**Table 3:** Risk factors for HCV.

Risk factors		HCV		*P* value^*∗*^
		No (*n* = 61)	Yes (*n* = 36)	
Age/years	Mean (SD)	7.69 (5.01)	12.63 (4.96)	0.04
	Median (range)	9 (1–19)	14 (6.5–18)	

Gender	Females	26 (42.62%)	9 (25.00%)	0.08
	Males	35 (57.38%)	27 (75.00%)	

Duration of the disease/years	Mean (SD)	7.75 (4.62)	10.56 (5.48)	0.02

Frequency of blood transfusion/months	Mean (SD)	1.11 (0.48)	1.04 (0.30)	0.57

Splenectomy	No	36 (59.02%)	20 (55.56%)	0.74
	Yes	25 (40.98%)	16 (44.44%)	

Ferritin level (ng/dL)	Mean (SD)	2416 (1283)	3652 (2173)	0.006

Alanine transaminase (ALT) (U/L)	Mean (SD)	48 (7.4)	65 (5.1)	0.001

Aspartate aminotransferase (AST) (U/L)	Mean (SD)	47 (9.44)	61 (8.9)	0.02

^*∗*^ *P* values were obtained by using Mann-Whitney test or Chi-square test.

**Table 4:** Risk factors for HBV.

Risk factors		HBV		*P* value^*∗*^
		No (*n* = 93)	Yes (*n* = 4)	
Age/years	Mean (SD)	7.77 (4.08%)	13.5 (1.73)	0.04
	Median (range)	8 (1–20)	13.5 (12–15)	

Gender	Females	34 (36.56%)	1 (25.00%)	0.08
	Males	59 (63.44%)	3 (75.00%)	

Duration of the disease/years	Mean (SD)	7.71 (4.86)	13.25 (1.73)	0.02

Frequency of blood transfusion/months	Mean (SD)	1.09 (0.43)	1 (0)	0.77

Splenectomy	No	56 (60.22%)	2 (50%)	1.00
	Yes	37 (39.78%)	2 (50%)	

Ferritin level (ng/dL)	Mean (SD)	2809 (1750)	4407 (1541)	0.04

Alanine transaminase (ALT) (U/L)	Mean (SD)	43 (4.4)	58 (3.5)	0.03

Aspartate aminotransferase (AST) (U/L)	Mean (SD)	42 (9.44)	63 (6.3)	0.04

^*∗*^ *P* values were obtained by using Mann-Whitney test or Chi-square test percentage.

**Table 5:** Risk factors for CMV.

Risk factors		CMV		*P* value^*∗*^
		No (*n* = 93)	Yes (*n* = 4)	
Age/years	Mean (SD)	8.69 (6.01)	13.63 (4.21)	0.03
	Median (range)	10 (1–21)	15 (6.5–18)	

Gender	Females	32 (34.41%)	3 (75.00%)	0.14
	Males	61 (65.59%)	1 (25.00%)	

Duration of the disease/years	Mean (SD)	7.82 (4.92)	13.69 (3.79)	0.03

Frequency of blood transfusion/months	Mean (SD)	1.09 (0.43)	0.88 (0.25)	0.21

Splenectomy	No	55 (59.14%)	1 (25.00%)	0.31
	Yes	38 (40.86%)	3 (75.00%)	

Ferritin level (ng/dL)	Mean (SD)	2894 (1770)	4735 (1789)	0.04

Alanine transaminase (ALT) (U/L)	Mean (SD)	55 (3.4)	56 (3.7)	0.43

Aspartate aminotransferase (AST) (U/L)	Mean (SD)	50 (6.44)	54 (6.8)	0.14

^*∗*^ *P* values were obtained by using Mann-Whitney test, Chi-square test, or Fisher exact test.
